# Ethyl 5-meth­oxy-2-trifluoro­methyl-1*H*-indole-3-carboxyl­ate

**DOI:** 10.1107/S1600536813002614

**Published:** 2013-02-02

**Authors:** Aurelien Crochet, Isak Alimi, Christian G. Bochet, Katharina M. Fromm

**Affiliations:** a Fribourg Center for Nanomaterials, FriMat, University of Fribourg, Chemin du Musee 3, CH-1700 Fribourg, Switzerland; b Chemistry Department, University of Fribourg, Chemin du Musee 9, CH-1700 Fribourg, Switzerland

## Abstract

The title compound, C_13_H_12_F_3_NO_3_, is almost planar if one excludes the F atoms of the –CF_3_ group [maximum deviation for the other hetero atoms = 0.069 (1) Å], and the dihedral angle between the pyrrole and benzene ring of the indole system is 2.54 (8)°. In the crystal, mol­ecules are linked by N—H⋯O hydrogen bonds, forming chains propagating along the *a-*axis direction. These chains are linked *via* C—H⋯O and C—H⋯F hydrogen bonds, forming a three-dimensional network.

## Related literature

For indoles, see: Kochanowska-Karamyan & Hamann (2010 ▶); Debieux & Bochet (2009 ▶); Helgen & Bochet (2003 ▶); Oppolzer *et al.* (1994 ▶), and for their synthesis, see: Chen *et al.* (2008 ▶); Barton *et al.* (1977 ▶). For photochemical methods for the synthesis of substituted indoles, see: Bochet & Blanc (2010 ▶); Bochet & Mercier (2009 ▶); Debieux & Bochet (2012 ▶); Streit & Bochet (2011 ▶); Alimi & Bochet (2013 ▶).
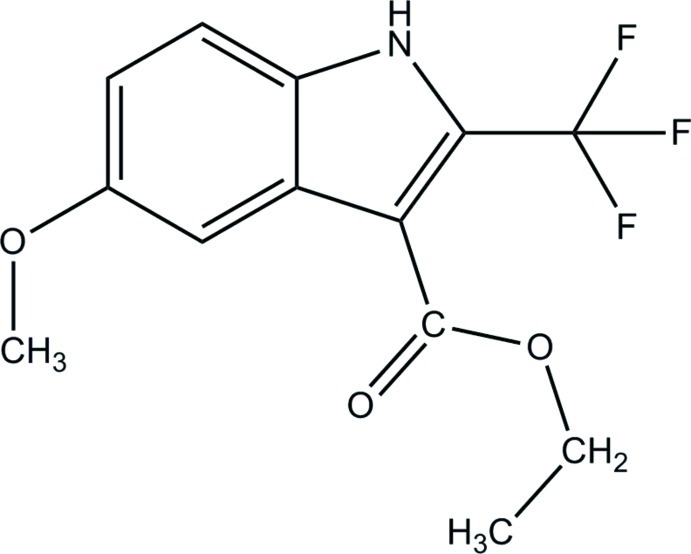


## Experimental

### 

#### Crystal data

C_13_H_12_F_3_NO_3_
*M* *_r_* = 287.24
Orthorhombic, 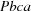

*a* = 13.9211 (8) Å
*b* = 8.6383 (4) Å
*c* = 21.5316 (7) Å
*V* = 2589.3 (2) Å^3^
*Z* = 8
Cu *K*α radiation
μ = 1.16 mm^−1^
*T* = 200 K
0.74 × 0.39 × 0.14 mm

#### Data collection

Stoe IPDS 2T diffractometer
Absorption correction: integration (*X-SHAPE*; Stoe & Cie, 2001 ▶) *T*_min_ = 0.610, *T*_max_ = 0.850
15525 measured reflections
2130 independent reflections
1894 reflections with *I* > 2σ(*I*)
*R*_int_ = 0.051

#### Refinement

*R*[*F* ^2^ > 2σ(*F* ^2^)] = 0.037
*wR*(*F* ^2^) = 0.097
*S* = 1.09
2130 reflections
186 parameters
H atoms treated by a mixture of independent and constrained refinement
Δρ_max_ = 0.19 e Å^−3^
Δρ_min_ = −0.19 e Å^−3^

### 

Data collection: *X-AREA* (Stoe & Cie, 2001 ▶); cell refinement: *X-AREA*; data reduction: *X-RED32* (Stoe & Cie, 2001 ▶); program(s) used to solve structure: *SHELXS97* (Sheldrick, 2008 ▶); program(s) used to refine structure: *SHELXL97* (Sheldrick, 2008 ▶); molecular graphics: *DIAMOND* (Brandenburg, 1999 ▶); software used to prepare material for publication: *publCIF* (Westrip, 2010 ▶).

## Supplementary Material

Crystal structure: contains datablock(s) I, global. DOI: 10.1107/S1600536813002614/su2553sup1.cif

Structure factors: contains datablock(s) I. DOI: 10.1107/S1600536813002614/su2553Isup2.hkl

Supplementary material file. DOI: 10.1107/S1600536813002614/su2553Isup3.cml

Additional supplementary materials:  crystallographic information; 3D view; checkCIF report

## Figures and Tables

**Table 1:** Hydrogen-bond geometry (Å, °)

*D*—H⋯*A*	*D*—H	H⋯*A*	*D*⋯*A*	*D*—H⋯*A*
N1—H1⋯O1^i^	0.835 (19)	2.109 (19)	2.8623 (16)	149.8 (16)
C6—H6⋯O3^ii^	0.95	2.53	3.460 (2)	168
C11—H11*B*⋯F2^iii^	0.99	2.50	3.336 (2)	142

Symmetry codes: (i) 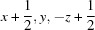; (ii) 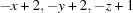; (iii) 
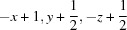
.

**Fig. 1.**
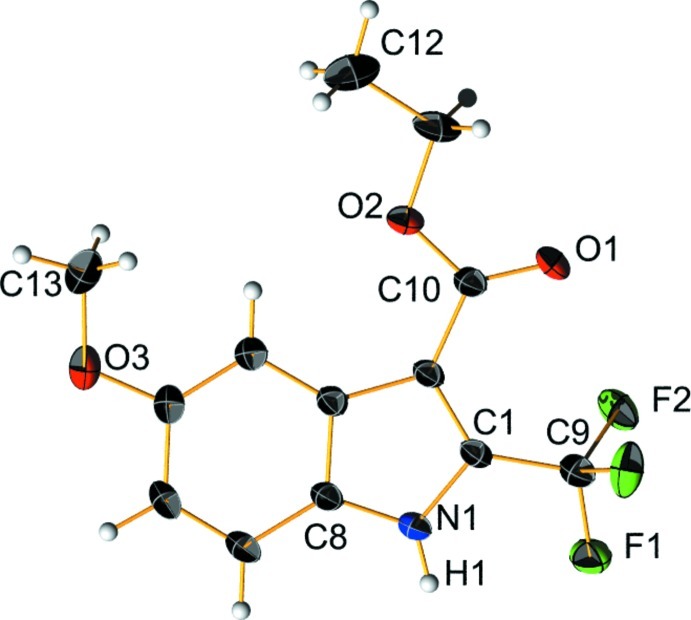
A view of the molecular structure of the title molecule, with atom labelling. The displacement ellipsoids are drawn at the 50% probability level.

**Fig. 2.**
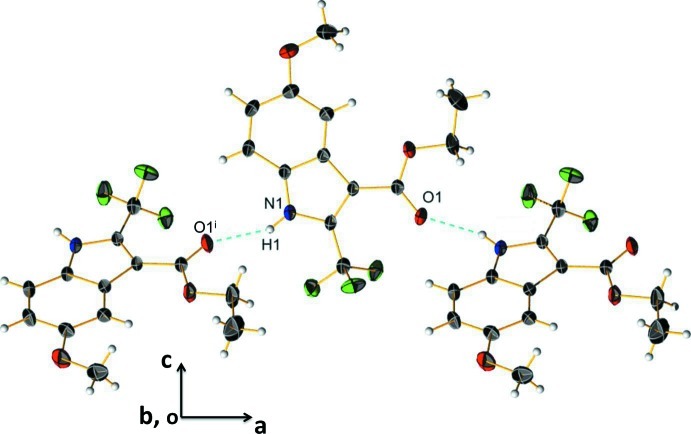
A view normal to the ac plane showing the N-H···O hydrogen bonded (dashed lines) chains propagating along the a axis direction in the crystal structure of the title compound [symmetry code: (i) x+1/2, y, -z+1/2; see Table 1 for details].

**Fig. 3.**
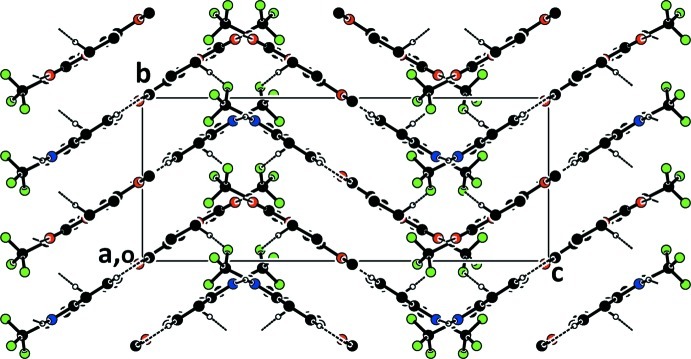
A view along the a axis of the crystal packing of the title compound. H atoms not involved in the C-H···O and C-H···F hydrogen bonds (dashed lines; see Table 1 for details) have been omitted for clarity.

## supplementary crystallographic information

### Comment

Indoles exhibit a plethora of biological applications (Kochanowska-Karamyan & Hamann, 2010), and a wide range of synthetic methods have been developed to synthesize them (Chen *et al.*, 2008; Barton *et al.*, 1977). Recently an efficient photochemical method for the synthesis of substituted indoles, based on a [3 + 2] cycloaddition of azide and alcynes, has been developed (Alimi & Bochet, 2013). During this work the title compound was synthesized and we report herein on its crystal structure.

The molecular structure of the title molecule is illustrated in Fig. 1. The molecule is almost planar [mean deviation for atoms (O1-O3,N1,C1-C8,C10-C13) is 0.069 (1) Å] with small deviations for the C9, CF_3_ group. The dihedral angle between the pyrrole (N1/C1-C3/C8) and the benzene (C3-C8) ring is only 2.54 (8)°.

In the crystal, N-H···O hydrogen bonds link the molecules to form chains propagating along the a axis direction (Table 1 and Fig. 2). These chains are linked via C-H···O and C-H···F hydrogen bonds forming a three-dimensional network (Table 1 and Fig. 3).

### Experimental

Ethyl 1-(4-methoxyphenyl)-5-(trifluoromethyl)-1*H*-1,2,3-triazole-4-carboxylate (101.7 g, 323 mmol) was dissolved in MeCN (15 ml) and irradiated during 10 h at 254 nm. The solution was concentrated *in vacuo* and purified with flash chromatography (Pentane: EtOAc, 4:1) to afford the title compound as a white solid [60.5 g, 211 mmol, 65.3% yield; M.p. 416 - 419 K; HRMS 310.0662 (C_13_H_12_F_3_NO_3_ + Na^+^; calcd. 310.0661]. The compound was recrystallized from chloroform to afford colourless block-like crystals suitable for X-ray analysis. Spectroscopic and other analytical data for the title compound are available in the archived CIF.

### Refinement

The NH H-atom was located in a difference Fourier map and refined with U_iso_(H) = 1.2*U*_eq_(N). The C-bound H-atoms were included in calculated positions and treated as riding atoms: C-H = 0.93, 0.97 and 0.96 Å for CH, CH_2_ and CH_3_ H-atoms, respectively, with U_iso_(H) = k × U_eq_(parent C-atom), where k = 1.5 for CH_3_ H-atoms and = 1.2 for other H-atoms.

### Crystal data

**Table:**

C_13_H_12_F_3_NO_3_	*D*_x_ = 1.474 Mg m^−^^3^
*M**_r_* = 287.24	Melting point: 416 K
Orthorhombic, *P**b**c**a*	Cu *K*α radiation, λ = 1.54186 Å
Hall symbol: -P 2ac 2ab	Cell parameters from 35241 reflections
*a* = 13.9211 (8) Å	θ = 3.2–67.3°
*b* = 8.6383 (4) Å	µ = 1.16 mm^−^^1^
*c* = 21.5316 (7) Å	*T* = 200 K
*V* = 2589.3 (2) Å^3^	Block, colourless
*Z* = 8	0.74 × 0.39 × 0.14 mm
*F*(000) = 1184	

### Data collection

**Table:**

Stoe IPDS 2T diffractometer	2130 independent reflections
Radiation source: fine-focus sealed tube	1894 reflections with *I* > 2σ(*I*)
Graphite monochromator	*R*_int_ = 0.051
Detector resolution: 6.67 pixels mm^-1^	θ_max_ = 65.0°, θ_min_ = 6.4°
rotation method scans	*h* = −16→16
Absorption correction: integration (*X-SHAPE*; Stoe & Cie, 2001)	*k* = −9→9
*T*_min_ = 0.610, *T*_max_ = 0.850	*l* = −25→25
15525 measured reflections	

### Refinement

**Table:**

Refinement on *F*^2^	Primary atom site location: structure-invariant direct methods
Least-squares matrix: full	Secondary atom site location: difference Fourier map
*R*[*F*^2^ > 2σ(*F*^2^)] = 0.037	Hydrogen site location: inferred from neighbouring sites
*wR*(*F*^2^) = 0.097	H atoms treated by a mixture of independent and constrained refinement
*S* = 1.09	*w* = 1/[σ^2^(*F*_o_^2^) + (0.0488*P*)^2^ + 1.0298*P*] where *P* = (*F*_o_^2^ + 2*F*_c_^2^)/3
2130 reflections	(Δ/σ)_max_ < 0.001
186 parameters	Δρ_max_ = 0.19 e Å^−^^3^
0 restraints	Δρ_min_ = −0.19 e Å^−^^3^

### Special details

**Table:**

Experimental. Spectroscopic data for the title compound: ^1^H NMR (360 MHz, CDCl_3_) δ p.p.m. 8.88 (br. s., 1 H) 7.74 (s, 1 H) 7.36 (d, *J* = 8.63 Hz, 1 H) 7.05 (d, *J* = 9.08 Hz, 1 H) 4.44 (q, *J* = 6.96 Hz, 2 H) 3.90 (s, 3 H) 1.44 (t, *J* = 7.04 Hz, 3 H). ^13^C NMR (75 MHz, CDCl_3_) d p.p.m. 163.48, 156.43, 129.48, 128.97, 128.84, 128.45, 127.94, 127.62, 125.75, 122.17, 118.60, 116.85, 115.03, 112.92, 107.77 (d, *J*=2.20 Hz, 1 C) 102.90, 60.57, 55.58, 14.05. IR (neat, cm^-1^) 3280, 2995, 2833, 1681, 1550, 1467, 1439, 1309, 1209, 1166, 1154, 1112, 1032, 845, 824, 713, 637. Rf 0.42 (Pentane: EtOAc 4:1).
Geometry. Bond distances, angles etc. have been calculated using the rounded fractional coordinates. All su's are estimated from the variances of the (full) variance-covariance matrix. The cell esds are taken into account in the estimation of distances, angles and torsion angles
Refinement. Refinement of F^2^ against ALL reflections. The weighted R-factor wR and goodness of fit S are based on F^2^, conventional R-factors R are based on F, with F set to zero for negative F^2^. The threshold expression of F^2^ > 2sigma(F^2^) is used only for calculating R-factors(gt) etc. and is not relevant to the choice of reflections for refinement. R-factors based on F^2^ are statistically about twice as large as those based on F, and R- factors based on ALL data will be even larger.

### Fractional atomic coordinates and isotropic or equivalent isotropic displacement parameters (Å^2^)

**Table:**

	*x*	*y*	*z*	*U*_iso_*/*U*_eq_	
F1	0.83489 (7)	0.47973 (13)	0.17707 (5)	0.0429 (3)	
F2	0.69256 (7)	0.43215 (11)	0.21010 (5)	0.0352 (3)	
F3	0.72136 (8)	0.64436 (11)	0.16278 (4)	0.0399 (3)	
O1	0.57084 (7)	0.64000 (15)	0.27202 (5)	0.0348 (4)	
O2	0.59337 (7)	0.76352 (14)	0.36252 (5)	0.0342 (4)	
O3	0.86605 (9)	0.98158 (18)	0.49087 (5)	0.0471 (5)	
N1	0.87955 (9)	0.64042 (15)	0.27672 (6)	0.0241 (4)	
C1	0.78495 (10)	0.61930 (17)	0.26378 (7)	0.0209 (4)	
C2	0.72915 (10)	0.68707 (17)	0.30968 (6)	0.0210 (4)	
C3	0.79547 (10)	0.75592 (18)	0.35320 (6)	0.0223 (4)	
C4	0.78425 (11)	0.8434 (2)	0.40791 (7)	0.0281 (5)	
C5	0.86630 (12)	0.8946 (2)	0.43723 (7)	0.0327 (5)	
C6	0.95870 (12)	0.8613 (2)	0.41420 (8)	0.0377 (6)	
C7	0.97083 (11)	0.7768 (2)	0.36098 (7)	0.0329 (5)	
C8	0.88812 (10)	0.72466 (18)	0.33081 (7)	0.0240 (4)	
C9	0.75746 (11)	0.54400 (17)	0.20418 (7)	0.0249 (4)	
C10	0.62433 (10)	0.69181 (18)	0.31147 (7)	0.0243 (4)	
C11	0.49061 (12)	0.7820 (3)	0.36956 (9)	0.0467 (7)	
C12	0.47534 (15)	0.8662 (3)	0.42942 (11)	0.0636 (9)	
C13	0.77531 (14)	1.0206 (3)	0.51655 (8)	0.0439 (6)	
H1	0.9254 (14)	0.616 (2)	0.2537 (8)	0.0290*	
H4	0.72230	0.86620	0.42400	0.0340*	
H6	1.01350	0.89810	0.43590	0.0450*	
H7	1.03300	0.75450	0.34520	0.0390*	
H11A	0.45860	0.67970	0.37070	0.0560*	
H11B	0.46390	0.84220	0.33450	0.0560*	
H12A	0.50170	0.80480	0.46370	0.0950*	
H12B	0.40640	0.88220	0.43600	0.0950*	
H12C	0.50790	0.96660	0.42770	0.0950*	
H13A	0.73750	1.07770	0.48570	0.0660*	
H13B	0.78460	1.08520	0.55350	0.0660*	
H13C	0.74110	0.92570	0.52820	0.0660*	

### Atomic displacement parameters (Å^2^)

**Table:**

	*U*^11^	*U*^22^	*U*^33^	*U*^12^	*U*^13^	*U*^23^
F1	0.0257 (5)	0.0536 (7)	0.0493 (6)	0.0019 (5)	0.0055 (4)	−0.0246 (5)
F2	0.0295 (5)	0.0287 (5)	0.0473 (6)	−0.0098 (4)	−0.0046 (4)	−0.0037 (4)
F3	0.0607 (7)	0.0331 (6)	0.0260 (5)	0.0016 (5)	−0.0127 (4)	0.0004 (4)
O1	0.0156 (5)	0.0566 (8)	0.0322 (6)	0.0001 (5)	−0.0052 (4)	−0.0080 (5)
O2	0.0154 (6)	0.0534 (7)	0.0339 (6)	0.0031 (5)	0.0021 (4)	−0.0119 (5)
O3	0.0381 (7)	0.0734 (10)	0.0299 (6)	−0.0043 (7)	−0.0059 (5)	−0.0191 (6)
N1	0.0140 (6)	0.0334 (7)	0.0249 (6)	0.0002 (5)	0.0015 (5)	−0.0004 (5)
C1	0.0145 (7)	0.0239 (8)	0.0242 (7)	−0.0004 (5)	−0.0021 (5)	0.0027 (6)
C2	0.0136 (7)	0.0270 (8)	0.0225 (7)	−0.0008 (6)	−0.0015 (5)	0.0030 (6)
C3	0.0176 (7)	0.0288 (8)	0.0204 (7)	−0.0012 (6)	−0.0018 (5)	0.0039 (6)
C4	0.0224 (8)	0.0403 (9)	0.0217 (7)	−0.0007 (7)	−0.0007 (6)	0.0009 (7)
C5	0.0294 (9)	0.0460 (10)	0.0227 (8)	−0.0045 (7)	−0.0047 (6)	−0.0033 (7)
C6	0.0233 (8)	0.0581 (12)	0.0318 (9)	−0.0098 (8)	−0.0086 (7)	−0.0034 (8)
C7	0.0167 (8)	0.0507 (11)	0.0313 (8)	−0.0062 (7)	−0.0029 (6)	0.0010 (7)
C8	0.0152 (7)	0.0338 (9)	0.0230 (7)	−0.0020 (6)	−0.0007 (6)	0.0025 (6)
C9	0.0197 (7)	0.0239 (8)	0.0310 (8)	0.0006 (6)	−0.0004 (6)	−0.0006 (6)
C10	0.0165 (7)	0.0326 (9)	0.0237 (7)	0.0008 (6)	−0.0002 (6)	0.0016 (6)
C11	0.0165 (8)	0.0685 (14)	0.0552 (12)	0.0071 (8)	0.0061 (7)	−0.0133 (10)
C12	0.0418 (12)	0.0882 (18)	0.0607 (14)	0.0105 (11)	0.0208 (10)	−0.0212 (12)
C13	0.0476 (11)	0.0566 (12)	0.0274 (9)	0.0025 (9)	0.0004 (7)	−0.0096 (8)

### Geometric parameters (Å, º)

**Table:**

F1—C9	1.3457 (18)	C4—C5	1.378 (2)
F2—C9	1.3289 (18)	C5—C6	1.408 (2)
F3—C9	1.3412 (18)	C6—C7	1.369 (2)
O1—C10	1.2150 (18)	C7—C8	1.397 (2)
O2—C10	1.3333 (19)	C11—C12	1.495 (3)
O2—C11	1.447 (2)	C4—H4	0.9500
O3—C5	1.378 (2)	C6—H6	0.9500
O3—C13	1.420 (2)	C7—H7	0.9500
N1—C1	1.3584 (19)	C11—H11A	0.9900
N1—C8	1.379 (2)	C11—H11B	0.9900
N1—H1	0.835 (19)	C12—H12A	0.9800
C1—C2	1.387 (2)	C12—H12B	0.9800
C1—C9	1.489 (2)	C12—H12C	0.9800
C2—C3	1.4437 (19)	C13—H13A	0.9800
C2—C10	1.460 (2)	C13—H13B	0.9800
C3—C8	1.403 (2)	C13—H13C	0.9800
C3—C4	1.408 (2)		
			
C10—O2—C11	117.20 (13)	F2—C9—C1	114.19 (13)
C5—O3—C13	117.27 (14)	O1—C10—O2	123.32 (13)
C1—N1—C8	109.15 (12)	O1—C10—C2	125.71 (14)
C8—N1—H1	124.7 (13)	O2—C10—C2	110.97 (12)
C1—N1—H1	125.8 (13)	O2—C11—C12	106.53 (15)
N1—C1—C9	118.99 (13)	C3—C4—H4	121.00
C2—C1—C9	130.91 (13)	C5—C4—H4	121.00
N1—C1—C2	109.89 (13)	C5—C6—H6	119.00
C3—C2—C10	127.62 (13)	C7—C6—H6	119.00
C1—C2—C3	106.16 (12)	C6—C7—H7	121.00
C1—C2—C10	126.19 (13)	C8—C7—H7	121.00
C4—C3—C8	119.51 (13)	O2—C11—H11A	110.00
C2—C3—C4	133.88 (13)	O2—C11—H11B	110.00
C2—C3—C8	106.59 (12)	C12—C11—H11A	110.00
C3—C4—C5	117.62 (14)	C12—C11—H11B	110.00
C4—C5—C6	122.02 (15)	H11A—C11—H11B	109.00
O3—C5—C4	123.85 (15)	C11—C12—H12A	109.00
O3—C5—C6	114.13 (14)	C11—C12—H12B	109.00
C5—C6—C7	121.08 (15)	C11—C12—H12C	109.00
C6—C7—C8	117.36 (14)	H12A—C12—H12B	110.00
N1—C8—C7	129.39 (13)	H12A—C12—H12C	110.00
C3—C8—C7	122.41 (14)	H12B—C12—H12C	110.00
N1—C8—C3	108.20 (12)	O3—C13—H13A	109.00
F1—C9—C1	110.38 (12)	O3—C13—H13B	110.00
F1—C9—F2	106.63 (12)	O3—C13—H13C	109.00
F1—C9—F3	106.17 (12)	H13A—C13—H13B	110.00
F3—C9—C1	112.76 (12)	H13A—C13—H13C	109.00
F2—C9—F3	106.20 (12)	H13B—C13—H13C	109.00
			
C11—O2—C10—O1	−1.4 (2)	C1—C2—C3—C8	−0.23 (16)
C11—O2—C10—C2	177.83 (15)	C10—C2—C3—C4	0.3 (3)
C10—O2—C11—C12	−179.41 (16)	C10—C2—C3—C8	−178.02 (14)
C13—O3—C5—C4	−0.5 (3)	C1—C2—C10—O1	−1.9 (3)
C13—O3—C5—C6	179.56 (16)	C1—C2—C10—O2	178.96 (14)
C8—N1—C1—C2	−0.98 (17)	C3—C2—C10—O1	175.48 (15)
C8—N1—C1—C9	174.28 (13)	C3—C2—C10—O2	−3.7 (2)
C1—N1—C8—C3	0.82 (17)	C2—C3—C4—C5	−178.23 (16)
C1—N1—C8—C7	−178.31 (16)	C8—C3—C4—C5	−0.1 (2)
N1—C1—C2—C3	0.74 (17)	C2—C3—C8—N1	−0.35 (17)
N1—C1—C2—C10	178.57 (14)	C2—C3—C8—C7	178.85 (14)
C9—C1—C2—C3	−173.78 (15)	C4—C3—C8—N1	−178.96 (14)
C9—C1—C2—C10	4.1 (3)	C4—C3—C8—C7	0.2 (2)
N1—C1—C9—F1	12.34 (19)	C3—C4—C5—O3	179.75 (15)
N1—C1—C9—F2	132.45 (14)	C3—C4—C5—C6	−0.3 (2)
N1—C1—C9—F3	−106.22 (16)	O3—C5—C6—C7	−179.55 (16)
C2—C1—C9—F1	−173.56 (15)	C4—C5—C6—C7	0.5 (3)
C2—C1—C9—F2	−53.5 (2)	C5—C6—C7—C8	−0.3 (2)
C2—C1—C9—F3	67.9 (2)	C6—C7—C8—N1	178.97 (16)
C1—C2—C3—C4	178.10 (17)	C6—C7—C8—C3	−0.1 (2)

### Hydrogen-bond geometry (Å, º)

**Table:**

*D*—H···*A*	*D*—H	H···*A*	*D*···*A*	*D*—H···*A*
N1—H1···O1^i^	0.835 (19)	2.109 (19)	2.8623 (16)	149.8 (16)
C6—H6···O3^ii^	0.95	2.53	3.460 (2)	168
C11—H11*B*···F2^iii^	0.99	2.50	3.336 (2)	142

Symmetry codes: (i) *x*+1/2, *y*, −*z*+1/2; (ii) −*x*+2, −*y*+2, −*z*+1; (iii) −*x*+1, *y*+1/2, −*z*+1/2.

## References

[bb1] Alimi, I. & Bochet, C. G. (2013). Unpublished data.

[bb2] Barton, D. H. R., Hesse, R. H., Jackman, G. P. & Pechet, M. M. (1977). *J. Chem. Soc. Perkin Trans. 1*, pp. 2604–2608.

[bb3] Bochet, C. G. & Blanc, A. (2010). In *Handbook of Synthetic Photochemistry*, edited by A. Albini & M. Fagnoni, pp. 417–447. Weinheim: Wiley-VCH.

[bb4] Bochet, C. G. & Mercier, S. (2009). In *Linker Strategies in Solid-Phase Organic Synthesis*, edited by P. Scott, pp. 151–193. New York: John Wiley.

[bb5] Brandenburg, K. (1999). *DIAMOND* Crystal Impact GbR, Bonn, Germany.

[bb6] Chen, Y., Wang, Y., Sun, Z. & Ma, D. (2008). *Org. Lett.* **4**, 625-628. 10.1021/ol7029382 18193883

[bb7] Debieux, J.-L. & Bochet, C. G. (2009). *J. Org. Chem.* **74**, 4519–4524. 10.1021/jo900442p 19476329

[bb8] Debieux, J.-L. & Bochet, C. G. (2012). *Chem. Sci.* **3**, 405–406.

[bb9] Helgen, C. & Bochet, C. G. (2003). *J. Org. Chem.* **68**, 2483–2486. 10.1021/jo026581n 12636422

[bb10] Kochanowska-Karamyan, A. J. & Hamann, M. T. (2010). *Chem. Rev.* **110**, 4489–4497. 10.1021/cr900211p PMC2922063 20380420

[bb11] Oppolzer, W., Spivey, A. C. & Bochet, C. G. (1994). *J.* *Am. Chem. Soc.* **116**, 3139–3140.

[bb12] Sheldrick, G. M. (2008). *Acta Cryst.* A**64**, 112–122. 10.1107/S0108767307043930 18156677

[bb13] Stoe & Cie (2001). *X-AREA*, *X-RED32* and *X-SHAPE* Stoe & Cie GmbH, Darmstadt, Germany.

[bb14] Streit, U. & Bochet, C. G. (2011). *Beilstein J. Org. Chem.* **7**, 525–542. 10.3762/bjoc.7.61 PMC3107530 21647263

[bb15] Westrip, S. P. (2010). *J. Appl. Cryst.* **43**, 920–925.

